# Effects of enhanced recovery after surgery-based nursing on clinical outcomes, psychological well-being, and quality of life in patients undergoing craniotomy for intracranial tumors

**DOI:** 10.3389/fneur.2026.1841041

**Published:** 2026-06-03

**Authors:** Ruitong Li, Jingqun Song, Junmei Xie

**Affiliations:** 1 Department of International Medical Service, Beijing Tiantan Hospital, Capital Medical University, Beijing, China; 2 Department of Medical Psychology for Elderly Children, Taonan Neuropsychiatric Hospital in Baicheng, Jilin, China; 3 Department of Endocrinology, People's Hospital of Peking University, Beijing, China

**Keywords:** craniotomy, enhanced recovery after surgery, intracranial tumors, perioperative nursing, quality of life

## Abstract

**Background:**

Intracranial tumor resection carries considerable risk and technical complexity. This study evaluates an Enhanced Recovery After Surgery (ERAS) nursing protocol on clinical recovery, psychological well-being, and quality of life.

**Methods:**

This prospective non-randomized comparative (quasi-experimental) study enrolled 162 patients undergoing elective craniotomy for intracranial tumors, allocated to an ERAS nursing group (EG; *n* = 81) or conventional care group (CG; *n* = 81) by admission ward. The primary outcome was length of hospital stay. Secondary outcomes included postoperative complications, recovery milestones, pain, in-hospital mortality, psychological well-being (Hamilton Anxiety [HAMA] and Depression [HAMD] Rating Scales), and health-related quality of life (SF-36), assessed preoperatively and on postoperative day 7. Between-group comparisons used unadjusted tests and multivariable models with prespecified covariates; the Benjamini–Hochberg procedure was applied to SF-36 domains.

**Results:**

Length of stay was shorter in the EG (9.2 ± 3.2 vs. 10.4 ± 3.8 days; mean difference −1.2 days, 95% CI −2.3 to −0.1; *p* = 0.031), and remained shorter after adjustment (−1.1 days, 95% CI −2.2 to −0.1; *p* = 0.038). Complications were less frequent in the EG (11.1% vs. 28.4%; unadjusted OR 0.31, 95% CI 0.13–0.72; *p* = 0.006; adjusted OR 0.34, 95% CI 0.14–0.81; *p* = 0.015). Times to first bowel movement, first defecation, and first solid food intake were all reduced (all *p* < 0.001), as was VAS pain at rest at 72 h (2.8 ± 1.2 vs. 4.6 ± 1.6; *p* < 0.001). On postoperative day 7, baseline-adjusted HAMA and HAMD scores were lower in the EG (*p* = 0.005 and *p* = 0.018), and SF-36 scores were higher across all eight domains and both component summaries (all Benjamini–Hochberg-adjusted *p* < 0.001).

**Conclusion:**

ERAS-based nursing was associated with shorter hospital stay, fewer complications, lower anxiety and depression, and higher quality of life after craniotomy for intracranial tumors. Given the non-randomized design, findings represent associations rather than causal effects; multicenter randomized validation with long-term follow-up is warranted.

## Introduction

Intracranial tumors are commonly encountered in neurosurgical practice and are classified as primary or secondary tumors (1). Primary tumors arise from the pituitary gland, meninges, neuroepithelial tissue, and hematopoietic organs (2). Secondary intracranial tumors are predominantly brain metastases, most frequently arising from lung cancer, melanoma, or renal carcinoma (3). Clinical manifestations vary according to the size and location of the lesion, with headache being the most common presentation; seizures and cognitive dysfunction may also occur (4). According to published epidemiological data, the incidence of brain tumors is approximately 6.4 per 100,000 person-years, with an overall five-year survival rate of 33.4% (5). Intracranial tumors are most prevalent between the ages of 55 and 64 years, with a slightly higher incidence in males (5). Surgical resection is the principal treatment for most intracranial tumors; however, the procedure carries considerable risks and technical complexities. Therefore, optimizing perioperative nursing care is essential to accelerate patient recovery, reduce postoperative stress responses, and minimize complications.

Enhanced recovery after surgery (ERAS) is a multimodal perioperative care pathway designed to reduce surgical stress and accelerate functional recovery. The concept was first proposed by Kehlet (6) and has since been widely adopted because of its demonstrated ability to reduce surgical complications, shorten hospital length of stay, and reduce patient financial burden. The ERAS framework has been applied in obstetrics and gynecology (7), general surgery (8), neurosurgery (9), and orthopedics (10), where it has achieved significant improvements in outcomes, including reduced perioperative stress responses and shortened intensive care unit length of stay. However, the effects of ERAS-based nursing protocols on anxiety, depression, and health-related quality of life in patients with intracranial tumors remain poorly characterized.

The present study describes a modified ERAS-based nursing protocol applied to craniotomy patients and evaluates its effects not only on the length of hospital stay and perioperative clinical outcomes but also on postoperative anxiety, depression, and quality of life, dimensions that have received insufficient attention in the existing neurosurgical ERAS literature. We hypothesized that, compared with conventional perioperative nursing, an ERAS-based nursing protocol would be associated with a shorter length of hospital stay (primary outcome) and reductions in postoperative complications, faster recovery milestones, lower postoperative pain, lower anxiety and depression scores, and higher health-related quality of life scores (secondary outcomes).

## Methodology

### Study design and setting

This prospective, non-randomized, comparative (quasi-experimental) study was conducted at Beijing Tiantan Hospital, Capital Medical University, Beijing, China. The study evaluated two pre-existing perioperative nursing pathways already in routine use in different wards within the same neurosurgery department; no patients were randomly assigned. This study was conducted in accordance with the principles of the Declaration of Helsinki and was approved by the Ethics Committee of Beijing Tiantan Hospital, Capital Medical University. Reporting follows the STROBE statement for observational studies, with additional reporting elements drawn from the TREND statement for non-randomized intervention studies. Because the comparison was based on the pre-existing ward-level standard of care rather than a prospectively assigned interventional protocol, the study was conducted as a quasi-experimental evaluation of two pre-existing nursing pathways and was not prospectively registered as a clinical trial; the authors acknowledge that prospective registration would have strengthened the design and recommend prospective registration for future ERAS evaluations of this type.

### Participants

One hundred and sixty-two patients who underwent elective craniotomy for intracranial tumors between January and December 2023 were enrolled. The inclusion criteria were as follows: (1) age ≥ 18 years; (2) confirmed indication for elective intracranial tumor surgery; and (3) ability to cooperate with treatment and complete assessments and provide informed consent. The exclusion criteria were as follows: (1) pre-existing psychiatric disorders, (2) significant cardiac, hepatic, or renal dysfunction, and (3) impaired consciousness or inability to cooperate with the assessment. Age, sex, BMI, ASA classification, comorbidities, and tumor type were recorded at enrolment.

**Figure 1:**
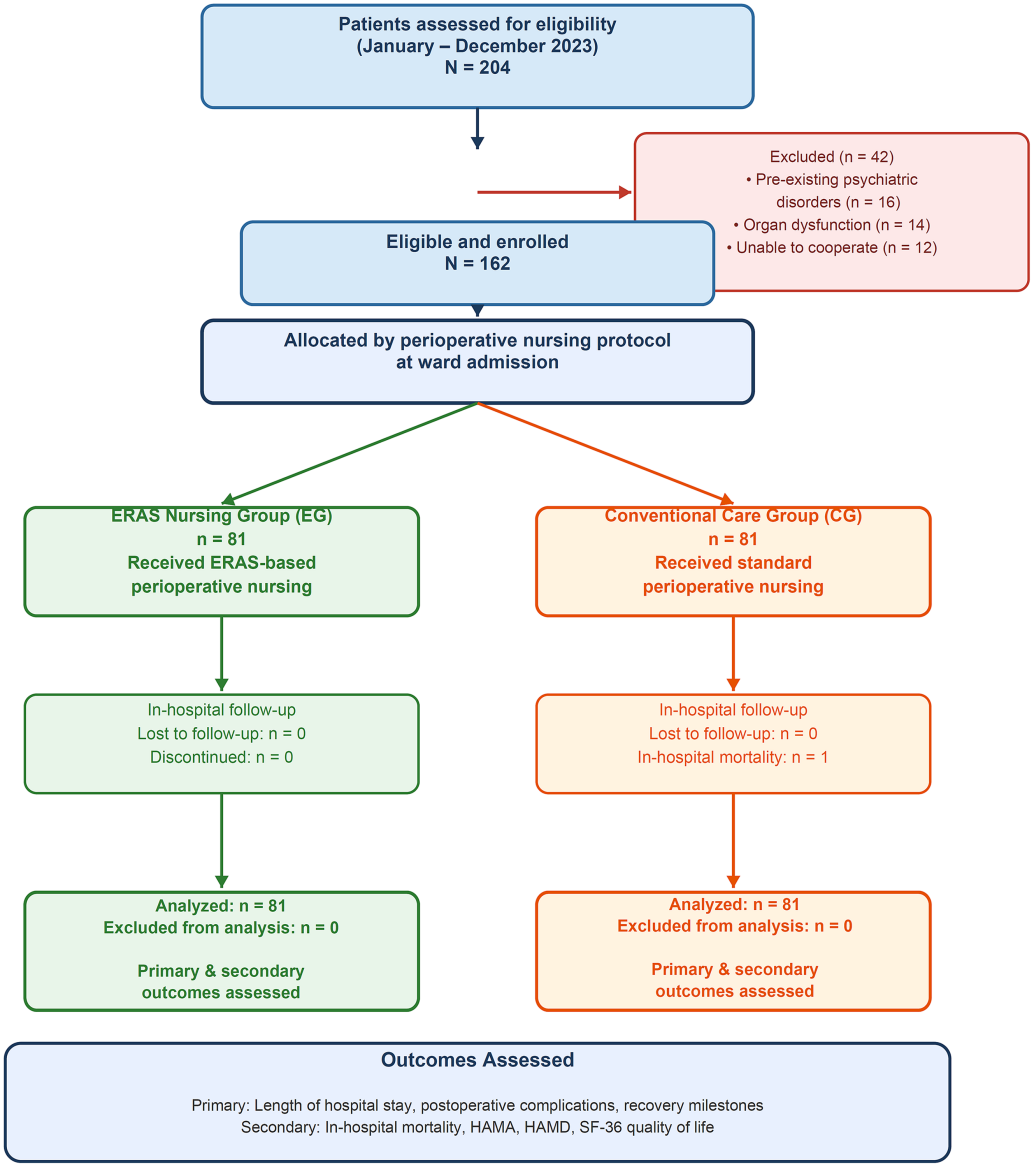
Patient flow diagram. A total of 204 patients were assessed for eligibility between January and December 2023. Forty-two patients were excluded due to pre-existing psychiatric disorders (*n* = 16), significant cardiac, hepatic, or renal dysfunction (*n* = 14), or inability to cooperate with assessment (*n* = 12). The remaining 162 patients were allocated to the ERAS nursing group (EG; *n* = 81) or the conventional care group (CG; *n* = 81) on the basis of the perioperative nursing protocol in routine use on the ward to which they were admitted. All 162 patients completed in-hospital follow-up and were included in the final analysis. ERAS, Enhanced Recovery After Surgery; EG, ERAS nursing group; CG, conventional care group; HAMA, Hamilton Anxiety Rating Scale; HAMD, Hamilton Depression Rating Scale; SF-36, 36-Item Short Form Health Survey.

Patients were divided into the ERAS nursing group (EG; *n* = 81) and conventional care group (CG; *n* = 81) based on the perioperative nursing protocol routinely used in the ward to which they were admitted. The two groups were managed concurrently throughout the study period (January–December 2023) in two separate wards of the same neurosurgery department, and no temporal cohort separation was used. Ward admission was determined administratively by bed availability and was not influenced by the patient, the patient’s family, the operating surgeon, or the nursing staff; neither the patient nor the surgical team selected the ward. The two wards were staffed by separate nursing teams with comparable clinical experience and nurse-to-patient ratios. Patients in both groups were operated upon by the same pool of attending neurosurgeons, with case-mix allocation overseen by the department. No major institutional, surgical, or anesthetic protocol changes were made during the study period. The two groups were managed in separate wards to minimize cross-group contamination. The patient flow through the study is illustrated in Figure 1. The sample size was determined *a priori* based on detecting a 1.5-day reduction in length of hospital stay with 80% power and alpha = 0.05, requiring 75 patients per group, which was rounded to 81 to account for potential study dropouts.

### Conventional care protocol

Patients in the CG received standard perioperative nursing care in routine use on the conventional ward at the time of the study, comprising the following components:

Preoperative care: Routine admission counseling was provided, with no structured psychological preparation or scripted preoperative information session. Preoperative fasting from solid food for 8–12 h and from clear liquids for at least 6 h was applied in accordance with the long-standing institutional fasting policy. No preoperative oral carbohydrate loading was given.

Intraoperative care: Intravenous fluid administration was at the discretion of the attending anesthesiologist with no protocol-mandated upper limit. Routine intraoperative warming was applied when clinically indicated.

Postoperative care:

(a) Dietary care: Diet was advanced from clear fluids to semi-solid food and then to regular food when the patient reported subjective tolerance without a scheduled stepwise protocol; intravenous fluid volumes were not routinely restricted.

(b) Mobilization care: Bed rest was maintained in the immediate postoperative period, and ambulation was initiated according to the patient’s clinical condition from postoperative day 1 onwards. The urinary catheter was routinely retained until the postoperative day 3.

(c) Pain care: Analgesia was administered parenterally on an as-needed basis based on physician order, without patient-controlled analgesia. Psychological support was provided informally as required, without a structured intervention. Standard discharge education was also provided.

Both groups received standard physician-directed medication administration, wound care, and patient monitoring. Therefore, the interventions differed in pathway content (timing and structure of nursing-led perioperative care) rather than in surgical or anesthetic techniques.

### ERAS-based nursing protocol

Patients in the EG received a structured nursing protocol guided by ERAS principles, comprising the following:

Preoperative care: Patients were visited by a dedicated nurse 1 day before surgery to assess their physical and psychological status, address individual concerns, and provide tailored guidance on managing anxiety and fear. A light diet was prescribed the day before surgery, avoiding spicy or irritating foods. Oral carbohydrate loading with 200–300 mL of 10% glucose solution was administered 3 h preoperatively, and fluid intake was restricted for 2 h before surgery.

Intraoperative care: Intravenous fluid administration was restricted to ≤20 mL/kg intraoperatively. Active warming measures were applied throughout the procedure to avoid hypothermia.

**Figure 2:**
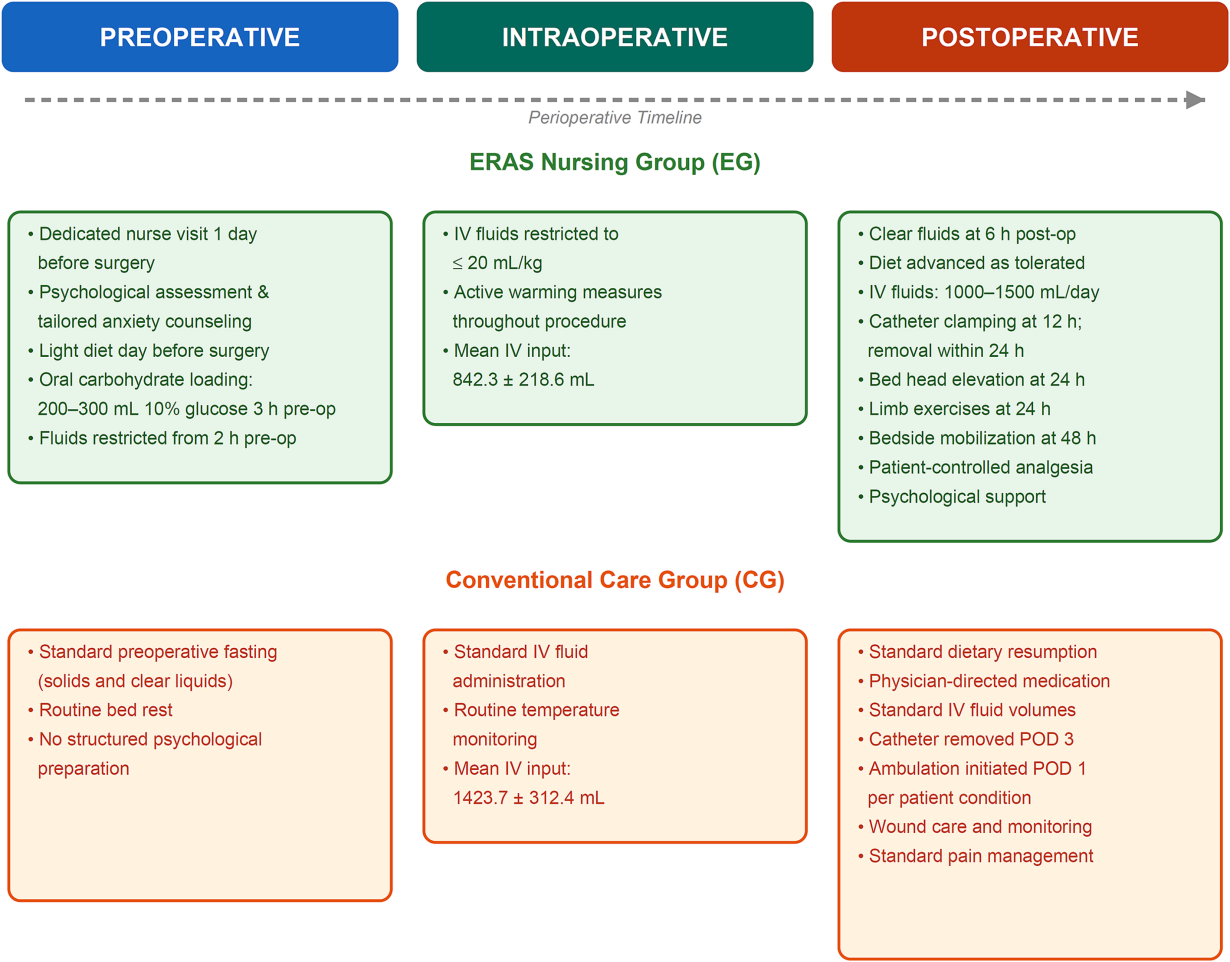
Overview of the ERAS-based nursing protocol compared with conventional perioperative care. The diagram summarizes the key interventions applied in the ERAS nursing group (EG) and conventional care group (CG) across three perioperative phases: preoperative, intraoperative, and postoperative. Principal differences include preoperative oral carbohydrate loading and structured psychological preparation, restricted intravenous fluid administration intraoperatively, and early dietary advancement, early catheter removal, structured mobilization, and patient-controlled analgesia postoperatively. ERAS, Enhanced Recovery After Surgery; IV, intravenous; POD, postoperative day.

Postoperative care:

(a) Dietary care: Clear fluids were permitted for 6 h after surgery. In the absence of discomfort, the diet was advanced to semisolid and then regular food as tolerated. Intravenous fluid volumes were restricted to 1,000–1,500 mL per day, limited to anti-vasospasm agents, and agents for intracranial pressure reduction.

(b) Mobilization care: Intermittent urinary catheter clamping training commenced 12 h postoperatively, and the catheter was removed within 24 h of surgery. The head of the bed was progressively elevated from postoperative hour 24. Simple active limb exercises in bed were initiated at 24 h, and patients were encouraged to mobilize at the bedside from 48 h postoperatively, with nursing staff providing supervised assistance.

(c) Pain care: Patient-controlled analgesia pumps were provided postoperatively, enabling self-titration of analgesic medication according to the pain intensity. Psychological support and meticulous wound care were provided as adjuncts to pharmacological analgesia. The key components of the ERAS-based nursing protocol, compared with conventional care, are shown in Figure 2.

### Outcome measures

The primary outcome was the length of hospital stay, defined as the number of calendar days from the day of surgery to the day of hospital discharge. The discharge criteria were prespecified and applied identically in both groups: (1) out-of-bed activity exceeding 6 h per day; (2) postoperative pain adequately controlled at rest (Visual Analog Scale [VAS] < 4); (3) recovery of bowel function with tolerance of solid food; and (4) absence of complications requiring continued hospitalization.

Secondary outcomes included postoperative complications, recovery milestones (time to first mobilization, time to first bowel movement, time to first defecation, and time to first solid food intake), postoperative pain (VAS score at rest at 72 h postoperatively), in-hospital mortality, psychological well-being assessed by the Hamilton Anxiety Rating Scale (HAMA) and Hamilton Depression Rating Scale (HAMD), and health-related quality of life assessed using the SF-36 questionnaire. The SF-36 individual domain scores were considered supportive secondary outcomes (see Statistical Analysis for multiplicity handling).

Complication definitions and ascertainment: Complications were prospectively documented during in-hospital follow-up only, from the time of surgery until hospital discharge. Operational definitions were applied uniformly across both groups: nausea and vomiting (any episode of clinically observed or patient-reported postoperative nausea or vomiting requiring antiemetic medication); hypothermia (intraoperative or immediate postoperative core temperature < 36.0 °C); pulmonary infection (clinical signs and chest imaging consistent with postoperative pneumonia, with positive sputum culture or treatment with antimicrobials based on attending physician judgment); deep vein thrombosis (lower-limb venous duplex ultrasound confirming thrombus formation); urinary tract infection (clinical symptoms with positive urinalysis or urine culture). A patient could contribute more than one complication category if multiple events occurred. The overall complication rate reported in Table 1 is the proportion of patients with at least one complication of any category. Complication ascertainment was conducted prospectively by attending nursing and medical staff and was not adjudicated by an independent, blinded committee. The operational definitions are summarized in Supplementary Table S1.

**Table 1:** Primary and key secondary outcome measures.

Variable	EG (*n* = 81)	CG (*n* = 81)	Mean difference (95% CI)	*p*-value
Length of hospital stay (days), mean ± SD (primary outcome)	9.2 ± 3.2	10.4 ± 3.8	−1.2 (−2.3 to −0.1)	0.031
Time to first mobilization (h), mean ± SD	50.2 ± 19.8	55.1 ± 23.4	−4.9 (−11.6 to 1.8)	0.146
Time to first bowel movement (h), mean ± SD	36.4 ± 8.2	52.8 ± 12.4	−16.4 (−19.7 to −13.1)	<0.001
Time to first defecation (h), mean ± SD	58.3 ± 14.6	84.7 ± 18.2	−26.4 (−31.5 to −21.3)	<0.001
Time to first solid food intake (h), mean ± SD	18.4 ± 4.8	31.6 ± 7.4	−13.2 (−15.1 to −11.3)	<0.001
VAS pain score at rest at 72 h postoperatively, mean ± SD	2.8 ± 1.2	4.6 ± 1.6	−1.8 (−2.2 to −1.4)	<0.001
Complications (any), *n* (%)	9 (11.1)	23 (28.4)	OR 0.31 (0.13 to 0.72)	0.006
Nausea and vomiting, *n* (%)	4 (4.9)	9 (11.1)	NA	NA
Hypothermia, *n* (%)	2 (2.5)	5 (6.2)	NA	NA
Pulmonary infection, *n* (%)	1 (1.2)	4 (4.9)	NA	NA
Deep vein thrombosis, *n* (%)	1 (1.2)	3 (3.7)	NA	NA
Urinary tract infection, *n* (%)	1 (1.2)	2 (2.5)	NA	NA

EG, ERAS nursing group; CG, conventional care group; VAS, visual analog scale; CI, confidence interval; OR = odds ratio. Student’s *t*-test for continuous variables; Pearson’s chi-square test (*χ*^2^ test: df = 1) for the overall complication rate. Per-category complication frequencies were presented descriptively only; no inferential between-group testing was performed at the individual complication level. The multivariate-adjusted estimates are presented in Supplementary Table S3.

Psychological and QoL assessments: HAMA, HAMD, and SF-36 were administered preoperatively at enrolment (within 24 h before surgery, after written informed consent) and postoperatively on postoperative day 7 (POD7), corresponding to a fixed assessment time point applied identically across both groups, irrespective of subsequent discharge timing. The SF-36 was scored according to the SF-36 Physical and Mental Health Component Scoring Manual (11, 12), using the validated Chinese-language version of the SF-36 with a one-week recall window appropriate for in-hospital postoperative use (13). Assessments were administered by two trained nurse-researchers who had received standardized training in HAMA, HAMD, and SF-36 administration prior to the study commencement. The same assessor administered the baseline and POD7 measurements for a given patient, where possible. Assessors were not formally blinded to group allocation because the structural differences between the two perioperative care pathways (e.g., presence of a patient-controlled analgesia pump, ward identity) made effective blinding infeasible. The SF-36 evaluates eight domains: Physical Functioning (PF), role-physical (RP), Bodily Pain (BP), General Health (GH), vitality (VT), Social Functioning (SF), role-emotional (RE), and Mental Health (MH). These eight domains are summarized as two component scores: the Physical Component Summary (PCS) and Mental Component Summary (MCS).

Protocol adherence: Adherence to the major components of the ERAS nursing protocol in the EG was prospectively documented in a standardized protocol adherence checklist completed by the ward nursing staff for each patient. The adherence rates for each major component are reported in Supplementary Table S2.

### Statistical analysis

Statistical analyses were performed using SPSS version 20.0 (IBM Corporation, Armonk, NY, United States) and R version 4.3.0 (R Foundation for Statistical Computing, Vienna, Austria). The normality of continuous variables was assessed using the Kolmogorov–Smirnov test. Normally distributed continuous variables are presented as mean ± standard deviation (SD) and compared between groups using Student’s independent-samples t-test (degrees of freedom = 160 for all between-group comparisons). Categorical variables are presented as frequencies (n) and percentages (%) and were compared using the Pearson chi-square test. Fisher’s exact test was applied when the expected cell counts were fewer than five. For ordinal variables (ASA classification), the chi-square test for trends was used. Effect sizes are reported alongside *p* values: Cohen’s d with 95% confidence intervals (CIs) for between-group differences in continuous outcomes and odds ratios (ORs) with 95% CIs for binary outcomes. For continuous outcomes, between-group mean differences with 95% confidence intervals (CIs) were also reported.

To address the non-randomized design and account for potential confounding factors, multivariable-adjusted analyses were performed for the primary and key secondary outcomes. Multivariable linear regression models were used for continuous outcomes (length of hospital stay, recovery milestones, and VAS pain score) with adjustment for the following prespecified covariates: age, sex, BMI, ASA class, presence of any comorbidity, tumor location category, surgery duration, intraoperative blood loss, and intraoperative intravenous fluid input. For HAMA, HAMD, and SF-36 outcomes, analysis of covariance (ANCOVA) was used, with the postoperative day 7 score as the dependent variable, group as the fixed factor, and the corresponding baseline (preoperative) score as a covariate, in addition to the pre-specified covariates listed above. Multivariable logistic regression was used for the binary complication outcome, with the same covariate set, and adjusted ORs with 95% CIs were reported. The adjusted analyses are presented in Supplementary Table S3.

To address the multiplicity arising from the eight SF-36 domain comparisons, the Benjamini–Hochberg false discovery rate procedure was applied to the eight SF-36 domain *p*-values; both unadjusted and Benjamini–Hochberg-adjusted *p*-values are reported. The two SF-36 component summary scores (PCS, MCS), HAMA, and HAMD were prespecified secondary endpoints and are reported without multiplicity correction; the increased risk of type I error from multiple secondary comparisons is acknowledged in the Discussion. All reported *p*-values were two-tailed, and statistical significance for the primary outcome was defined as *p* < 0.05. The single in-hospital death in the conventional care group occurred prior to the postoperative day 7 assessment; therefore, that patient was not included in the postoperative day 7 HAMA, HAMD, or SF-36 analyses, as reported in the Results section. All other patients completed the planned in-hospital assessment.

## Results

### Baseline characteristics

There were no statistically significant differences in the baseline demographic or clinical characteristics between the two groups (Table 2). The groups were comparable with respect to age (EG: 52.4 ± 12.3 years vs. CG: 54.1 ± 13.1 years; *t*-test: df = 160; *p* = 0.374), BMI (EG: 23.8 ± 3.2 kg/m^2^ vs. CG: 23.8 ± 3.4 kg/m^2^; *t*-test: df = 160; *p* = 0.967), sex distribution (male: 54.3% vs. 51.9%; *χ*^2^ test: df = 1; *p* = 0.752), ASA classification (*p* = 0.384), and prevalence of individual comorbidities (all *p* > 0.05).

**Table 2:** Baseline characteristics of 162 patients.

Variable	EG (*n* = 81)	CG (*n* = 81)	*p*-value
Age (years), mean ± SD	52.4 ± 12.3	54.1 ± 13.1	0.374
Gender, male, *n* (%)	44 (54.3)	42 (51.9)	0.752
BMI (kg/m^2^), mean ± SD	23.8 ± 3.2	23.8 ± 3.4	0.967
ASA classification, *n* (%)			0.384
Class I	15 (18.5)	14 (17.3)	
Class II	51 (63.0)	49 (60.5)	
Class III	15 (18.5)	18 (22.2)	
Comorbidities, *n* (%)			
Hypertension	22 (27.2)	24 (29.6)	0.726
Diabetes mellitus	12 (14.8)	14 (17.3)	0.660
Cardiac disease	8 (9.9)	9 (11.1)	0.793

EG, ERAS nursing group; CG, conventional care group; BMI, body mass index; ASA, American Society of Anesthesiologists. Student’s *t*-test was used for continuous variables, Pearson’s chi-square test for binary categorical variables, and the chi-square test for trend for ASA classification. The baseline preoperative HAMA, HAMD, and SF-36 scores are presented in Table 3.

Baseline preoperative HAMA, HAMD, and SF-36 scores were comparable between the two groups, with no statistically significant differences (Table 3). Baseline HAMA scores were 15.6 ± 4.2 in the EG vs. 15.4 ± 4.5 in the CG (*p* = 0.756), baseline HAMD scores were 14.2 ± 4.6 vs. 14.0 ± 5.0 (*p* = 0.789), and baseline SF-36 PCS and MCS scores were 56.8 ± 12.4 vs. 57.1 ± 12.9 (*p* = 0.881) and 54.3 ± 13.6 vs. 54.0 ± 13.9 (*p* = 0.892), respectively. The baseline scores for the individual SF-36 domains and the corresponding between-group *p*-values are reported in Table 3.

**Table 3:** Secondary outcome measures, including in-hospital mortality and psychological/quality-of-life assessments at baseline and on postoperative day 7.

Variable	EG (*n* = 81)	CG (*n* = 81)	*p*-value
In-hospital mortality, *n* (%)	0 (0.0)	1 (1.2)	>0.999
Baseline (preoperative) scores, mean ± SD			
HAMA score	15.6 ± 4.2	15.4 ± 4.5	0.756
HAMD score	14.2 ± 4.6	14.0 ± 5.0	0.789
SF-36 PF	58.4 ± 14.2	59.1 ± 13.8	0.752
SF-36 RP	52.6 ± 17.8	53.1 ± 18.2	0.860
SF-36 BP	57.2 ± 16.4	56.8 ± 17.1	0.879
SF-36 GH	52.1 ± 15.6	52.8 ± 16.0	0.778
SF-36 VT	50.3 ± 14.2	50.7 ± 14.6	0.860
SF-36 SF	58.6 ± 14.8	58.2 ± 15.3	0.866
SF-36 RE	53.4 ± 19.2	53.0 ± 19.8	0.896
SF-36 MH	55.1 ± 14.0	54.7 ± 14.4	0.857
SF-36 PCS	56.8 ± 12.4	57.1 ± 12.9	0.881
SF-36 MCS	54.3 ± 13.6	54.0 ± 13.9	0.892
Postoperative day 7 scores, mean ± SD			
HAMA score	8.3 ± 3.6	10.4 ± 4.8	0.002
HAMD score	8.2 ± 4.8	10.3 ± 5.6	0.011
SF-36 PF	74.3 ± 12.6	61.8 ± 14.2	<0.001
SF-36 RP	68.2 ± 16.4	54.7 ± 18.3	<0.001
SF-36 BP	72.4 ± 14.8	58.9 ± 17.2	<0.001
SF-36 GH	64.8 ± 15.2	53.6 ± 16.4	<0.001
SF-36 VT	62.4 ± 13.8	51.2 ± 15.6	<0.001
SF-36 SF	71.6 ± 14.2	60.8 ± 16.3	<0.001
SF-36 RE	65.3 ± 18.6	52.4 ± 20.1	<0.001
SF-36 MH	66.8 ± 13.4	55.7 ± 15.8	<0.001
SF-36 PCS	69.9 ± 11.8	57.3 ± 13.6	<0.001
SF-36 MCS	66.5 ± 13.2	55.0 ± 15.3	<0.001

EG, ERAS nursing group; CG, conventional care group; HAMA, Hamilton Anxiety Rating Scale; HAMD, Hamilton Depression Rating Scale; PF, Physical Functioning; RP, Role-Physical; BP, Bodily Pain; GH, General Health; VT, Vitality; SF, Social Functioning; RE, Role-Emotional; MH, Mental Health; PCS, Physical Component Summary; MCS, Mental Component Summary. Student’s *t*-test was used for continuous variables, and Fisher’s exact test was used for mortality. The single in-hospital death in the CG occurred before postoperative day 7 and was excluded from the postoperative day 7 HAMA, HAMD, and SF-36 analyses (postoperative day 7 analyses therefore included 81 EG and 80 CG patients). Baseline-adjusted (ANCOVA) and multivariable-adjusted estimates with 95% CIs and Benjamini–Hochberg-adjusted *p*-values for the eight SF-36 domain comparisons are reported in Supplementary Table S3.

### Intraoperative parameters

The intraoperative characteristics and tumor location/lesion category distributions are summarized in Table 4. Surgery duration (EG: 285.3 ± 62.4 min vs. CG: 291.7 ± 68.1 min; *t*-test: df = 160; *p* = 0.527), intraoperative blood loss (EG: 312.4 ± 124.6 mL vs. CG: 328.7 ± 138.2 mL; *t*-test: df = 160; *p* = 0.413), intraoperative temperature (EG: 36.42 ± 0.48 °C vs. CG: 36.38 ± 0.52 °C; *t*-test: df = 160; *p* = 0.614), and urine output (EG: 1124.6 ± 342.8 mL vs. CG: 1183.5 ± 378.4 mL; *t*-test: df = 160; *p* = 0.283) were comparable between groups, with no statistically significant differences. Intraoperative intravenous fluid input was significantly lower in the EG than in the CG (842.3 ± 218.6 mL vs. 1423.7 ± 312.4 mL; *t*-test: df = 160; *p* < 0.001). The distribution of the tumor anatomical region/lesion category was similar across groups, with sellar/suprasellar lesions being the most common category in both groups (*p* = 0.962). The categories reported in Table 4 are a mixture of anatomical regions and lesion/pathology descriptors, reflecting how the cases were recorded in the source dataset.

**Table 4:** Intraoperative parameters and tumor location/lesion category in EG and CG.

Variable	EG (*n* = 81)	CG (*n* = 81)	*p*-value
Surgery duration (min), mean ± SD	285.3 ± 62.4	291.7 ± 68.1	0.527
Blood loss (mL), mean ± SD	312.4 ± 124.6	328.7 ± 138.2	0.413
Intraoperative temperature (°C), mean ± SD	36.42 ± 0.48	36.38 ± 0.52	0.614
Urine output (mL), mean ± SD	1124.6 ± 342.8	1183.5 ± 378.4	0.283
Intravenous fluid input (mL), mean ± SD	842.3 ± 218.6	1423.7 ± 312.4	< 0.001
Tumor location/lesion category, *n* (%)			0.962
CPA region	14 (17.3)	13 (16.0)	
Sellar/suprasellar region	18 (22.2)	17 (21.0)	
Frontal/insular region	16 (19.8)	15 (18.5)	
Corpus callosum region	8 (9.9)	9 (11.1)	
Clivus/chordoma	5 (6.2)	6 (7.4)	
Cerebellar region	12 (14.8)	13 (16.0)	
Cavernous hemangioma	8 (9.9)	8 (9.9)	

EG, ERAS nursing group; CG, conventional care group; CPA, cerebellopontine angle. Student’s *t*-test for continuous variables; chi-square test for the distribution of tumor location/lesion category. The categories listed are a mixture of anatomical regions and lesion/pathology descriptors as recorded in the source dataset; detailed histopathologic diagnoses, tumor size, supra- vs. infratentorial classification, and skull-base vs. non-skull-base categorization were not available.

### Primary outcome

The primary outcome data are presented in Table 1. The length of hospital stay was significantly shorter in the EG than in the CG (9.2 ± 3.2 days vs. 10.4 ± 3.8 days; mean difference −1.2 days, 95% CI − 2.3 to −0.1; Cohen’s d = 0.34, 95% CI 0.03 to 0.65; t-test: df = 160; *p* = 0.031). After multivariable adjustment for age, sex, BMI, ASA class, comorbidities, tumor location category, surgery duration, blood loss, and intraoperative intravenous fluid input, the difference remained statistically significant (adjusted mean difference −1.1 days, 95% CI − 2.2 to −0.1; *p* = 0.038).

### Secondary outcomes

The secondary outcome data are presented in Tables 1, 3. The times to first bowel movement (36.4 ± 8.2 h vs. 52.8 ± 12.4 h; *t*-test: df = 160; *p* < 0.001), first defecation (58.3 ± 14.6 h vs. 84.7 ± 18.2 h; *t*-test: df = 160; *p* < 0.001), and first solid food intake (18.4 ± 4.8 h vs. 31.6 ± 7.4 h; *t*-test: df = 160; *p* < 0.001) were all significantly shorter in the EG. The postoperative VAS pain scores at rest at 72 h were significantly lower in the EG (2.8 ± 1.2 vs. 4.6 ± 1.6; *t*-test: df = 160; *p* < 0.001). The time to first mobilization showed a non-significant trend toward earlier mobilization in the EG (50.2 ± 19.8 h vs. 55.1 ± 23.4 h; *t*-test: df = 160; *p* = 0.146). The direction and statistical significance of these comparisons were preserved after multivariate adjustment (Supplementary Table S3).

The overall complication rate (proportion of patients with at least one complication of any category) was significantly lower in the EG (9/81 [11.1%] vs. 23/81 [28.4%]; unadjusted OR 0.31, 95% CI 0.13–0.72; *χ*^2^ test: df = 1; *p* = 0.006). After multivariable adjustment, the association persisted (adjusted OR 0.34, 95% CI 0.14–0.81; *p* = 0.015). The most frequent complication in both groups was nausea and vomiting. Full details of the complication categories are provided in Table 1; per-category *p*-values are provided as descriptive information only, with the prespecified inferential comparison being the overall complication rate.

There was no significant difference in in-hospital mortality between the groups (EG: 0/81 [0.0%] vs. CG: 1/81 [1.2%]; Fisher’s exact test: *p* > 0.999). Given that this represents a single event in one group only, this comparison should be interpreted with caution and is not informative for between-group comparisons. The single deceased patient was excluded from the postoperative day 7 HAMA, HAMD, and SF-36 analyses, as the assessment time point was not reached.

After adjusting for baseline (preoperative) values via ANCOVA, postoperative day 7 HAMA scores were significantly lower in the EG than in the CG (8.3 ± 3.6 vs. 10.4 ± 4.8; baseline-adjusted mean difference −2.0, 95% CI −3.4 to −0.6; *p* = 0.005), as were the HAMD scores (8.2 ± 4.8 vs. 10.3 ± 5.6; baseline-adjusted mean difference −1.9, 95% CI −3.4 to −0.4; *p* = 0.018).

Postoperative day 7 SF-36 scores were significantly higher in the EG across all eight domains, both before and after adjustment for baseline values via ANCOVA. Unadjusted between-group differences (with Benjamini–Hochberg-corrected *p*-values) were as follows: Physical Functioning (74.3 ± 12.6 vs. 61.8 ± 14.2; *p* < 0.001; BH-adjusted *p* < 0.001), Role-Physical (68.2 ± 16.4 vs. 54.7 ± 18.3; *p* < 0.001; BH-adjusted *p* < 0.001), Bodily Pain (72.4 ± 14.8 vs. 58.9 ± 17.2; *p* < 0.001; BH-adjusted *p* < 0.001), General Health (64.8 ± 15.2 vs. 53.6 ± 16.4; *p* < 0.001; BH-adjusted *p* < 0.001), Vitality (62.4 ± 13.8 vs. 51.2 ± 15.6; *p* < 0.001; BH-adjusted *p* < 0.001), Social Functioning (71.6 ± 14.2 vs. 60.8 ± 16.3; *p* < 0.001; BH-adjusted *p* < 0.001), Role-Emotional (65.3 ± 18.6 vs. 52.4 ± 20.1; *p* < 0.001; BH-adjusted *p* < 0.001), and Mental Health (66.8 ± 13.4 vs. 55.7 ± 15.8; *p* < 0.001; BH-adjusted *p* < 0.001). Both SF-36 component summary scores were significantly higher in the EG: PCS (69.9 ± 11.8 vs. 57.3 ± 13.6; *p* < 0.001) and MCS (66.5 ± 13.2 vs. 55.0 ± 15.3; *p* < 0.001). Baseline-adjusted (ANCOVA) and multivariable-adjusted estimates with 95% CIs are reported in Supplementary Table S3, and the direction and significance of the SF-36 group differences were preserved across all sensitivity analyses.

### Protocol adherence

Adherence to the major components of the ERAS nursing protocol was high. Among the 81 patients in the EG, the proportions of those receiving the major protocol components were as follows: structured preoperative nursing visit and psychological preparation, 81/81 (100%); preoperative oral carbohydrate loading (200–300 mL of 10% glucose 3 h preoperatively), 78/81 (96.3%); intraoperative intravenous fluid restriction (≤20 mL/kg), 76/81 (93.8%); intraoperative active warming, 81/81 (100%); urinary catheter removal within 24 h postoperatively, 75/81 (92.6%); structured early mobilization (in-bed exercises at 24 h, bedside mobilization from 48 h), 73/81 (90.1%); and patient-controlled analgesia provision, 79/81 (97.5%). The component-level adherence is summarized in Supplementary Table S2. Fidelity in the conventional care group was not formally documented.

## Discussion

The application of ERAS pathways in neurosurgery remains less established than in other surgical specialties, with a relative lack of supporting data. The primary objective of this study was to evaluate the effects of an ERAS-based nursing protocol on clinical recovery outcomes, complication rates, psychological well-being, and quality of life in patients who underwent craniotomy for intracranial tumors.

Conventional perioperative nursing practice has historically advocated extended preoperative fasting (14), whereas the ERAS concept advocates modified fasting protocols adapted to the patient’s specific clinical context. In the present study, the ERAS protocol incorporated solid food fasting from 6–10 h preoperatively, with clear fluid restriction from 2–3 h before surgery and preoperative oral carbohydrate loading (200–300 mL of 10% glucose solution) administered 3 h before surgery. Intraoperative intravenous fluid volumes were restricted and continued to be minimized postoperatively (15). Published evidence indicates that appropriately timed preoperative oral intake does not increase the risk of aspiration, nausea, or vomiting and helps maintain energy substrate availability, thereby supporting gastrointestinal recovery (16). Controlled perioperative fluid administration has been shown to reduce the risk of postoperative hypoglycemia and insulin resistance, the latter being a recognized contributor to prolonged hospital stay (17, 18). In the present study, intraoperative intravenous fluid input was significantly lower in the EG than in the CG (842.3 ± 218.6 mL vs. 1423.7 ± 312.4 mL; *p* < 0.001), which was associated with reduced complication rates and shorter length of hospital stay.

Encouraging early postoperative mobilization is a central component of the ERAS pathway. In the present study, the nursing staff provided structured verbal and practical guidance on early mobilization and rehabilitation to reduce psychological barriers and promote physical recovery. Although the difference in time to first mobilization did not reach statistical significance (50.2 ± 19.8 h vs. 55.1 ± 23.4 h; *p* = 0.146), the observed trend was consistent with the direction of effect reported in the ERAS literature. Chen et al. (19) found that early ambulation guided by ERAS principles promoted bowel function and shortened hospital stay in patients undergoing laparoscopic myomectomy. Teeuwen et al. (20) reported a reduction in length of stay from 9 to 6 days in patients who underwent colorectal surgery and were managed under the ERAS protocol. Elayat et al. (21) found that ERAS-based care had no significant effect on the length of stay in patients undergoing elective craniotomy, possibly because early ambulation protocols were not detailed in that study. In the present study, the times to first bowel movement, first defecation, and first solid food intake were all significantly shorter in the EG than in the CG (all *p* < 0.001), and the complication rate was significantly lower (*χ*^2^ test: df = 1; *p* = 0.006).

Postoperative pain after craniotomy is clinically significant, with published data indicating that up to 60% of patients experience moderate-to-severe pain in the early postoperative period (22, 23). Uncontrolled pain impairs recovery and adversely affects the patient’s quality of life (24). The patient-controlled analgesia protocol implemented in the EG, combined with psychological support and wound care, was associated with significantly lower VAS pain scores at rest 72 h postoperatively compared with the CG (2.8 ± 1.2 vs. 4.6 ± 1.6; *p* < 0.001).

Patient psychological well-being and quality of life are important dimensions of perioperative care quality, and their inclusion as secondary outcomes in this study adds to the existing literature, which has largely focused on the length of stay and complication rates. Liu et al. (25) demonstrated that ERAS protocols shortened hospital stay and improved patient satisfaction in elective craniotomy. León et al. (26) found improvements in quality of life measures among colorectal cancer patients managed with ERAS protocols. In the present study, baseline-adjusted HAMA and HAMD scores on postoperative day 7 were significantly lower in the EG than in the CG (*p* = 0.005 and *p* = 0.018, respectively), and SF-36 scores were significantly higher across all eight domains (all Benjamini–Hochberg-adjusted *p* < 0.001). These improvements likely reflect the combined contributions of superior pain control, reduced complication rates, shorter hospital stay, and structured psychological support embedded within the ERAS nursing protocol.

These findings are consistent with the broader emerging neuro-oncology literature emphasizing that psychological burden, treatment satisfaction, and health-related quality of life are critical patient-centered outcomes in neurosurgical and brain tumor populations. Patients undergoing neurosurgery for brain metastases or other intracranial tumors carry a substantial psycho-oncological burden, including anxiety, depression, and impaired health-related quality of life, both perioperatively and across the longer-term illness trajectory (27–30). Routine assessment of patient-centered outcomes, including standardized HRQoL instruments, has been advocated as essential for surgical neuro-oncology care, and unmet psychosocial needs are common in both patients and family caregivers, supporting the integration of structured psychological support and HRQoL assessment into perioperative pathways, such as the one described here.

Several alternative explanations for the observed differences in psychological well-being and QoL should be considered. First, the EG received structured preoperative psychological preparation and continuous nursing-led psychological support, which differs from the informal psychological support available in the CG. The observed differences in HAMA and HAMD scores may therefore partly reflect this differential intensity of psychological care rather than the broader ERAS protocol. Second, the EG had significantly better postoperative pain control, with markedly lower 72-h VAS scores; given the well-established bidirectional relationship between postoperative pain and psychological symptoms, improved analgesia likely contributed to the reduction in HAMA and HAMD scores. Third, the EG experienced fewer in-hospital complications and earlier resumption of bowel function and oral intake, which may secondarily improve patient-reported well-being and SF-36 domain scores, irrespective of the ERAS pathway as a whole. Fourth, because postoperative HAMA, HAMD, and SF-36 assessments were administered on postoperative day 7 by assessors who were not formally blinded to group allocation, the possibility of expectation and assessor bias cannot be excluded from the study. These considerations support a cautious interpretation of the observed psychological and QoL differences and underline the need for randomized, blinded-outcome-assessment validation in future studies.

Although the broader ERAS literature spans multiple surgical specialties, neurosurgery-specific evidence on ERAS pathways remains comparatively limited and is dominated by single-center studies (21, 25). Compared with previously published neurosurgical ERAS evaluations, this study contributes additional data on patient-reported psychological and quality-of-life outcomes, which represent dimensions that have been recognized as a priority in surgical neuro-oncology but are infrequently reported in neurosurgical ERAS studies. Direct comparison with other neurosurgical ERAS evaluations is limited by heterogeneity in protocol composition, control care intensity, outcome definitions, and patient mix; however, the directions of effect observed here for length of stay, recovery milestones, and complications are broadly consistent with prior neurosurgical reports (31–33).

This study has several important limitations that should be considered when interpreting its findings. First, the design was non-randomized: patients were allocated to the ERAS or conventional care pathway by ward of admission rather than by a randomized procedure. Although ward admission was determined administratively and was not influenced by patients, surgeons, or nursing staff, the absence of randomization introduces the possibility of selection bias and ward-level confounding (e.g., differences in nursing team experience, ward resources, or unmeasured case-mix factors), which multivariable adjustment can only partially mitigate. Second, although baseline preoperative HAMA, HAMD, and SF-36 assessments were performed and were comparable between groups, residual confounding by unmeasured baseline differences cannot be excluded. Third, the SF-36 individual domain comparisons involved multiple statistical tests, which increases the risk of type I error; although the Benjamini–Hochberg procedure was applied, this only partially addresses multiplicity, and SF-36 domain results should be regarded as supportive rather than confirmatory evidence. Fourth, although secondary outcome assessments were administered at a fixed postoperative time point (postoperative day 7), assessors were not formally blinded to group allocation, raising the possibility of expectation and assessor bias for the subjective patient-reported outcomes (pain, anxiety, depression, quality of life). Fifth, the study population represents a heterogeneous group of intracranial tumors, and the data set did not include detailed histopathologic diagnoses, tumor size, supratentorial vs. infratentorial classification, skull-base vs. non-skull-base categorization, eloquent-area involvement, surgical approach, or extent of resection; these are recognized determinants of perioperative outcomes and quality of life and may have contributed to outcome heterogeneity that we were unable to fully adjust for in multivariable models. Sixth, although protocol adherence in the ERAS group was prospectively documented and was high, fidelity in the conventional care group was not formally assessed; some unmeasured cross-contamination of practices between wards cannot be entirely excluded. Seventh, follow-up was limited to in-hospital assessment, with the postoperative day 7 time point as the latest measurement; the sustained effect of the ERAS-based nursing pathway on long-term recovery, mental health, and quality of life after discharge is not addressed. Eighth, the study was conducted at a single tertiary referral center, which may limit generalizability to other healthcare settings, patient populations, and resource environments. Finally, in-hospital mortality was based on a single event in one group, which is too sparse to support meaningful between-group comparison.

## Conclusion

In the present study, an ERAS-based nursing protocol was associated with a shorter postoperative length of hospital stay, lower in-hospital complication rates, lower postoperative anxiety and depression scores, and higher health-related quality-of-life scores in patients undergoing craniotomy for intracranial tumors. Given the non-randomized design, ward-based allocation, and methodological limitations described above, these findings should be interpreted as associations rather than causal effects of the ERAS protocol. Multicenter randomized evaluations with formal blinding of outcome assessors and longer post-discharge follow-ups are warranted to validate these findings, determine their generalizability across diverse healthcare settings, and clarify the sustained impact of ERAS-based nursing on patient recovery and well-being. Subject to these caveats, the present findings contribute to the optimization of perioperative nursing care in this patient population.

## Data Availability

The original contributions presented in the study are included in the article/Supplementary material, further inquiries can be directed to the corresponding author.
