# Supplementary material for: Effects of enhanced recovery after surgery-based nursing on clinical outcomes, psychological well-being, and quality of life in patients undergoing craniotomy for intracranial tumors
Source: Front Neurol. 2026 Jun 3;17:1841041. doi: 10.3389/fneur.2026.1841041 (PMC13272006; doi:10.3389/fneur.2026.1841041)
Supplement: Supplementary file 1 [file Table_1.DOCX]

**Supplementary Table S1. Operational definitions of in-hospital postoperative complications.**

| **Complication** | **Operational definition** | **Ascertainment** |
| --- | --- | --- |
| Nausea and vomiting | Any episode of clinically observed or patient-reported postoperative nausea or vomiting requiring antiemetic medication. | Prospectively documented by ward nursing staff during in-hospital follow-up. |
| Hypothermia | Intraoperative or immediate postoperative core temperature < 36.0°C, measured by core temperature probe (intraoperative) or tympanic thermometer (postoperative). | Prospectively documented from anesthetic and post-anesthesia care unit records. |
| Pulmonary infection | Clinical signs (productive cough, fever, abnormal auscultation) plus chest imaging consistent with postoperative pneumonia, with positive sputum culture or initiation of antimicrobial therapy on attending physician judgment. | Prospectively documented during in-hospital follow-up. |
| Deep vein thrombosis | Lower-limb venous duplex ultrasound demonstrating thrombus formation, performed when clinically suspected. | Prospectively documented during in-hospital follow-up. |
| Urinary tract infection | Clinical symptoms (urinary frequency, dysuria, suprapubic pain, fever) with positive urinalysis (significant pyuria) or positive urine culture. | Prospectively documented during in-hospital follow-up. |

All complications were captured during the in-hospital period only (from the time of surgery to hospital discharge). Complications were documented prospectively by attending nursing and medical staff and were not adjudicated by an independent blinded committee. A patient could contribute to more than one complication category if multiple events occurred. The overall complication rate reported in Table 4 is the proportion of patients with at least one complication of any category.

**Supplementary Table S2. Adherence to major components of the ERAS nursing protocol in the ERAS group (n = 81).**

| **ERAS protocol component** | **Patients receiving the component, n (%)** |
| --- | --- |
| Structured preoperative nursing visit and psychological preparation | 81/81 (100.0) |
| Preoperative oral carbohydrate loading (200–300 mL of 10% glucose, 3 hours preoperatively) | 78/81 (96.3) |
| Intraoperative intravenous fluid restriction (≤ 20 mL/kg) | 76/81 (93.8) |
| Intraoperative active warming | 81/81 (100.0) |
| Postoperative clear fluids initiated within 6 hours of surgery | 79/81 (97.5) |
| Restricted postoperative intravenous fluid volume (1000–1500 mL/day) | 77/81 (95.1) |
| Urinary catheter removal within 24 hours postoperatively | 75/81 (92.6) |
| Structured early mobilization (in-bed exercises at 24 h, bedside mobilization from 48 h) | 73/81 (90.1) |
| Patient-controlled analgesia provision | 79/81 (97.5) |

Adherence was prospectively documented in a standardized protocol-adherence checklist completed by the ward nursing staff for each patient. Non-adherence reflects predominantly clinical contraindications documented in the patient record (e.g., delayed mobilization for neurological deterioration, withholding of carbohydrate loading for diabetic patients on insulin, prolonged catheterization for urinary retention or fluid balance monitoring). Fidelity in the conventional care group was not formally documented.

**Supplementary Table S3. Multivariable-adjusted analyses for primary and key secondary outcomes.**

| **Outcome** | **Unadjusted estimate (95% CI)** | **Adjusted estimate (95% CI)†** | **Adjusted P-value** |
| --- | --- | --- | --- |
| Length of hospital stay (days), mean difference (EG − CG) | −1.2 (−2.3 to −0.1) | −1.1 (−2.2 to −0.1) | 0.038 |
| Time to first mobilization (h), mean difference | −4.9 (−11.6 to 1.8) | −4.4 (−10.9 to 2.1) | 0.184 |
| Time to first bowel movement (h), mean difference | −16.4 (−19.7 to −13.1) | −15.9 (−19.2 to −12.6) | < 0.001 |
| Time to first defecation (h), mean difference | −26.4 (−31.5 to −21.3) | −25.7 (−30.7 to −20.7) | < 0.001 |
| Time to first solid food intake (h), mean difference | −13.2 (−15.1 to −11.3) | −12.9 (−14.8 to −11.0) | < 0.001 |
| VAS pain score at rest at 72 h, mean difference | −1.8 (−2.2 to −1.4) | −1.7 (−2.1 to −1.3) | < 0.001 |
| Any complication, OR (EG vs. CG) | 0.31 (0.13 to 0.72) | 0.34 (0.14 to 0.81) | 0.015 |
| HAMA score at POD7, mean difference‡ | −2.1 (−3.4 to −0.8) | −2.0 (−3.4 to −0.6) | 0.005 |
| HAMD score at POD7, mean difference‡ | −2.1 (−3.7 to −0.5) | −1.9 (−3.4 to −0.4) | 0.018 |
| SF-36 PF at POD7, mean difference‡ | 12.5 (8.4 to 16.6) | 12.0 (8.0 to 16.0) | < 0.001 (BH < 0.001) |
| SF-36 RP at POD7, mean difference‡ | 13.5 (8.2 to 18.8) | 12.9 (7.7 to 18.1) | < 0.001 (BH < 0.001) |
| SF-36 BP at POD7, mean difference‡ | 13.5 (8.5 to 18.5) | 13.0 (8.1 to 17.9) | < 0.001 (BH < 0.001) |
| SF-36 GH at POD7, mean difference‡ | 11.2 (6.3 to 16.1) | 10.7 (5.9 to 15.5) | < 0.001 (BH < 0.001) |
| SF-36 VT at POD7, mean difference‡ | 11.2 (6.7 to 15.7) | 10.8 (6.4 to 15.2) | < 0.001 (BH < 0.001) |
| SF-36 SF at POD7, mean difference‡ | 10.8 (6.0 to 15.6) | 10.4 (5.7 to 15.1) | < 0.001 (BH < 0.001) |
| SF-36 RE at POD7, mean difference‡ | 12.9 (7.0 to 18.8) | 12.4 (6.6 to 18.2) | < 0.001 (BH < 0.001) |
| SF-36 MH at POD7, mean difference‡ | 11.1 (6.6 to 15.6) | 10.6 (6.2 to 15.0) | < 0.001 (BH < 0.001) |
| SF-36 PCS at POD7, mean difference‡ | 12.6 (8.5 to 16.7) | 12.1 (8.1 to 16.1) | < 0.001 |
| SF-36 MCS at POD7, mean difference‡ | 11.5 (7.0 to 16.0) | 11.0 (6.6 to 15.4) | < 0.001 |

EG = ERAS nursing group; CG = conventional care group; OR = odds ratio; CI = confidence interval; VAS = Visual Analogue Scale; POD7 = postoperative day 7; HAMA = Hamilton Anxiety Rating Scale; HAMD = Hamilton Depression Rating Scale; PF = Physical Functioning; RP = Role-Physical; BP = Bodily Pain; GH = General Health; VT = Vitality; SF = Social Functioning; RE = Role-Emotional; MH = Mental Health; PCS = Physical Component Summary; MCS = Mental Component Summary; BH = Benjamini–Hochberg-adjusted (false discovery rate) P value, applied to the eight SF-36 domain comparisons. †Adjusted analyses: multivariable linear regression for continuous outcomes and multivariable logistic regression for the binary complication outcome, with adjustment for age, sex, BMI, ASA class, presence of any comorbidity, tumor location category, surgery duration, intraoperative blood loss, and intraoperative intravenous fluid input. ‡ANCOVA models additionally adjusted for the corresponding baseline (preoperative) score.
